# Local hyperthermia in head and neck cancer: mechanism, application and advance

**DOI:** 10.18632/oncotarget.10350

**Published:** 2016-06-30

**Authors:** Shiyu Gao, Min Zheng, Xiaohua Ren, Yaling Tang, Xinhua Liang

**Affiliations:** ^1^ State Key Laboratory of Oral Diseases West China Hospital of Stomatology, Sichuan University, Chengdu, China; ^2^ Department of Oral and Maxillofacial Surgery, Affiliated Hospital of Stomatology, Nanjing Medical University, Nanjing, China; ^3^ Department of Stomatology, Zhoushan Hospital, Zhoushan, China; ^4^ Department of Stomatology, Sichuan Medical Science Academy and Sichuan Provincial People's Hospital, Chengdu, China; ^5^ Department of Oral and Maxillofacial Surgery West China Hospital of Stomatology, Sichuan University, Chengdu, China

**Keywords:** local hyperthermia, head and neck cancer, heat shock proteins (HSPs), nanoparticle, near-infrared (NIR)

## Abstract

Local hyperthermia (HT), particularly in conjunction with surgery, radiotherapy and chemotherapy was useful for the treatment of human malignant tumors including head and neck cancer. However, at present it suffered from many limitations such as thermal dose control, target treatment regions and discrimination between healthy and cancer cells. Recent developments in nanotechnology have introduced novel and smart therapeutic nanomaterials to local HT of head and neck cancer that basically take advantage of various targeting approaches. The aim of this paper is to give a brief review of the mechanism, methods and clinical applications of local HT in head and neck cancer, mainly focusing on photothermal therapy (PTT) and nanoparticle-based hyperthermia.

## INTRODUCTION

Head and neck cancer (HNC) refers to a group of malignancies which are divided into seven anatomic sites, lip, oral, maxillary sinus, pharynx, salivary gland, larynx and thyroid, respectively. It represents the sixth most prevalent cancer worldwide. The 5-year survival rate is about 50% for all sites and stages [1]. Despite advancements in chemotherapy, radiotherapy and surgical therapy, there has been little improvement in survival rates over the past 50 years [2].

Hyperthermia (HT), especially local HT, offers a promising prospect for the treatment of head and neck cancer. It is characterized by treatment of a wide range of lesions with minimal adverse effect and adjacent tissue damage, and thus allowed good functional and aesthetic results [3]. HT has been used for cancer therapy for more than 5,000 years. The first provable report was in the Egyptian Edwin Smith surgical papyrus, which was dated around 3000 BC [4]. It was later forgotten until the end of the 19th century, when the deep penetrating energy transfer was solved through electromagnetism. [5]. In the last several decades, due to the interdiscipline application of the physics, engineering and biology, HT developed by leaps and bounds [6].

Now a days, hyperthermia has been regarded as the fifth treatment of cancer including surgery, chemotherapy, radiotherapy and immunotherapy. It normally contributes to improve clinical response and reduce toxicities to radiotherapy and chemotherapy [7]. However, the trimodality (thermochemoradiation), in which radiotherapy, chemotherapy and hyperthermia are combined, has not yet been formally established in clinical application. The main obstacle is the limitation of the current techniques on accurate positioning and temperature equilibrium control. Recently, with the development of nanotechnology, photothermal therapy (PTT) shows a promising approach [3], which starts a resurgence of hyperthermia. Here, the article will systematically retrospect the mechanisms, methods of and the application of the local HT in head and neck cancer. The development of PTT and nanoparticle-based hyperthermia will be discussed.

## MECHANISMS OF LOCAL HYPER-THERMIA

**Figure 1:**
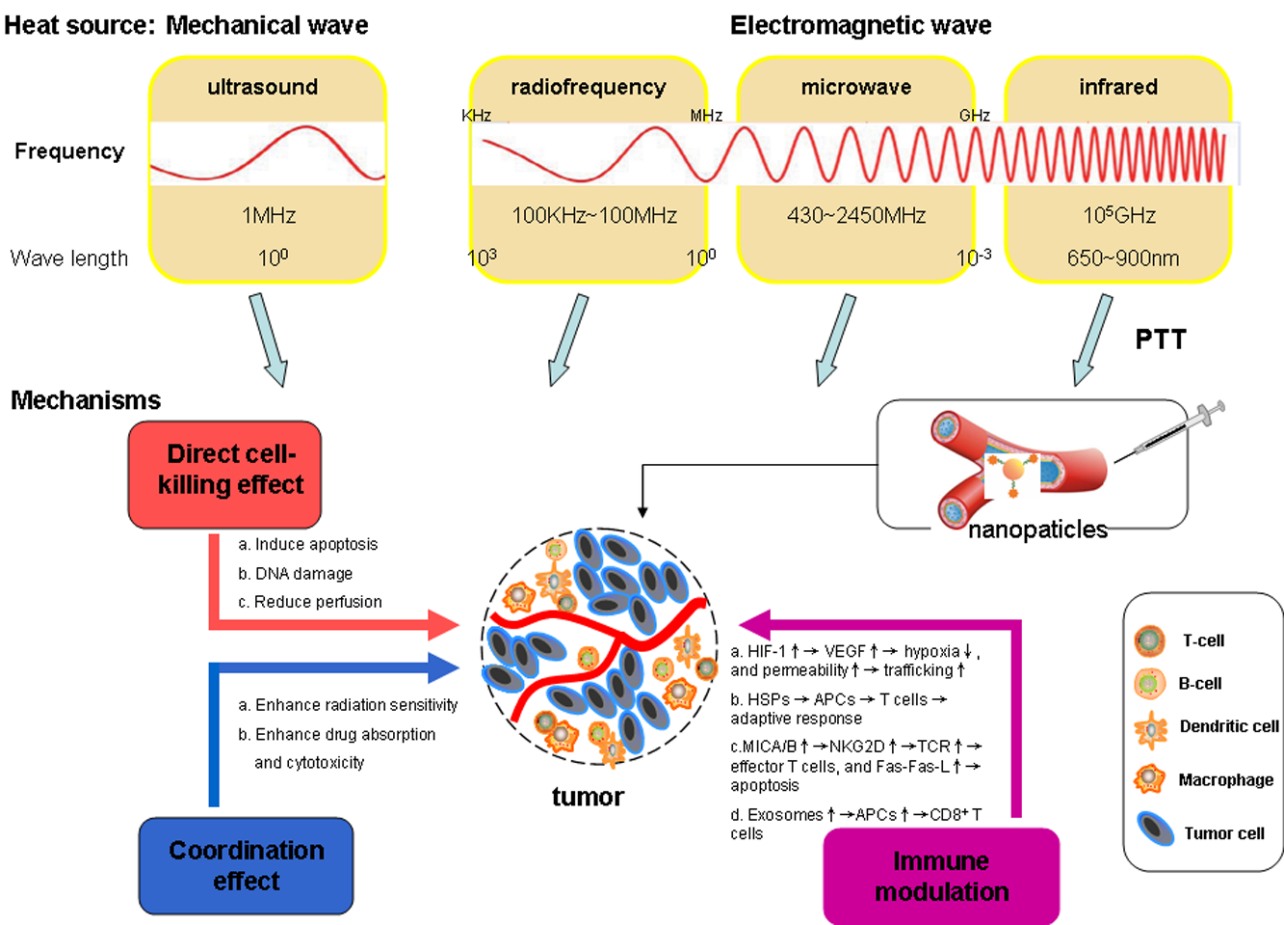
Heat sources used for hyperthermia purposes and different mechanisms induced by locally heating tumors The top half of figure shows major heat sources, divided by different physical frequencies and properties, which are ultrasound, microwave and near-infrared (NIR), respectively. The energy is converted into heat by means of photothermal nanoparticles. The mechanism of killing tumor cells *via* the following aspects: (1) Direct cell-killing effects of HT alone (red); (2) Anti-neoplastic effect coordinated with radiotherapy and chemotherapy (blue); (3) Immune modulation (purple).

Local HT is a therapy that selectively heats the tumor to treatment temperature (39~45°C) by physical heating device, which can kill cancer cells but not injure normal cells (Figure 1). The mechanism of local HT mainly includes the following aspects:

### The direct cell-killing effects

To individual cancer cells, heating could change cell membrane permeability and receptors, alter enzyme activity and cellular structure, to induce cell apoptosis [2]. Exposure of cells to heating causes a rapid translocation of nucleolin from the nucleolus into the nucleoplasm, which inhibits DNA replication and synthesis. Cells in the synthesis phase (S phase) suffered the heating impact the most seriously, due to the damage of chromatin structure and inactivation of replication protein [8]. There are five major factors in intratumor microenvironment such as perfusion, permeability, pO_2_, pH and pressure to greatly affect the response of tumors to HT [9, 10].

In tongue squamous cell line Tca8113, Jiang et al. [11] showed that phosphorylation of PLS3 by PKC-delta was involved in the hyperthermia-induced apoptotic signal pathway and that HSP27 blocked this pathway to suppress hyperthermia-induced apoptosis. To confirm the detailed molecular mechanism underlying cell death induced by local HT, Tabuchi et al. [12] examined the gene expression patterns and gene networks in oral squamous cell carcinoma (OSCC) HSC-3 cells using a combination of DNA microarray and bioinformatics tools. Microarray analysis revealed that 14 genes such as ATF3, DUSP1 and JUN, were associated with relevant biological functions including cell death and cellular movement, and 13 genes such as BAG3, DNAJB1 and HSPA1B, were associated with cellular function, maintenance and cellular assembly, and organization. The expression of apoptosis inhibitory proteins such as Bcl-2, Bcl-xL, NF-kappaB, COX2, STAT3, IL-6, and IKKalpha/1 was also highly induced after heat treatment of OSCC by protein microarray analysis [13].

### Anti-neoplastic effect coordinated with radiotherapy and chemotherapy

Heating after irradiating significantly can reduce the dose of radiation therapy. HT is a potent radiation sensitizer [14]. Moderate HT leads to increased vascular permeability and increase in oxygen pressure levels in the tumors. This altered microenvironment due to HT enhances the radiosensitivity of the tumor. Thermal radiation sensitization may also be due to DNA inhibition of repair and alteration in nuclear protein aggregation and higher order chromatin organization [15]. Higuchi et al. [16] found that the effects of thermoradiotherapy in all 8 squamous cell carcinoma (SCC) cell lines was improved using recombinant p53-expressing adenovirus.

To chemotherapy, heating probably changes the permeability of cell membrane to enhance drug absorption and alters drug metabolism to improve cytotoxic effect. Moreover, it has an active effect on tumor cells of chemotherapy drug resistance [8]. Sato et al. [17] found that the combination therapy for oralcancerwith cisplatin andhyperthermia generated with ferucarbotran in an alternating magnetic field (AMF) might reduce the clinically effective dosage of cisplatin. Further, Sato et al.[18] found that the combined hyperthermia-chemotherapy with magnetically guided Fe nanoparticles to treat tongue cancer in a rabbit model dramatically reduced the tumor masses, which represented a powerful new approach for head and neck cancer. However, not all drugs have shown adequate thermal enhancement. Vinca alkaloids, taxanes, 5-FU and methotrexate are unsatisfactory to add the thermal effect *in vitro* studies [19].

### Hypoxia

Local HT could contribute to changing tumor vessel perfusion and pO_2_, through activating HIF-1 and its downstream targets, such as VEGF and pyruvate dehydrogenase kinase 1(PDK1), and modifying tumor cell metabolism signaling pathways [20]. Moreover, local HT can enhance the permeability of tumor vasculature [21], then convert vessels to high-rate trafficking sites, and finally facilitate recruiting of immune cells (i.e., natural killer [NK] cells, CD8+ T cells, and neutrophils) into tumor tissues. Winslow et al. [22] found that wild systemic heating maintained at 39.5 ± 0.5 °C for 4 h can significantly alter the tumor microenvironment of human head and neck tumor xenograft models, decreasing interstitial fluid pressure (IFP), and hypoxia while increasing microvascular perfusion and radiosensitivity.

### Heat-shock proteins (HSPs)

The acquisition of thermotolerance in cancer cells renders hyperthermia less effective. Heat-stressed tumor cells release heat-shock proteins (HSPs), including HSP27, HSP70, HSP90, which can bind to and activate antigen-presenting cells (APCs) in the next step [23, 24]. Once HSPs chaperone cancer antigens is phagocytosed by APCs [25], the complex will be transported to T cells to initiate adaptive immune responses [26, 27].

Yunoki et al. [28] demonstrated that the sensitivity to HT (44 °C, 90 min) was remarkably enhanced in HSC-3 cells with silencing BAG3, a co-chaperone of the heat shock protein 70. There was a positive correlation between DNA binding (Id-1) expression and the expression of p-Akt, p-GSK3β and p-HSF1 in 76 OSCC patients. The inhibition of Id-1 expression can improve the efficacy of hyperthermiain OSCC [29]. Oba et al. [30] demonstrated that IFN-gamma suppressed HSP27 basal transcription and promoter activity specifically through one of the two Sp1 sites in the proximal region of the HSP27 promoter in OSCC HSC-2. And the combination treatment of hyperthermia and IFN-gamma suppressed tumor growth *in vivo* more effectively than hyperthermia alone. Heating of humanoral cancercell lines HSC4 cells at 45 °C for 20 min gradually increased H3-Lys4 and H3-Lys9 methylation. The induction of HSPs by heating may be correlated with at heat-induced methylation of histone H3 [31].

### Anti-tumor immunity

A great deal of attention has been focused on the ability and molecular mechanism of HT to regulate the anti-tumor immunity system. HT alters the visibility of tumors to immune cells *via* increasing cytotoxic potential. MHC class I ligand (MICA/B), on the surface of tumor cells, were overexpressed when heated at 39.5 to 45 °C, and then activated the receptor NKG2D (Natural killer group 2, member D) on NKs or CD8^+^ T cells [32, 33]. NKG2D on NK cells could directly induce cytotoxicity, and the expression of NKG2D^+^ CD8^+^ on T-cells can costimulate T-cell receptor (TCR) signaling [34], overcome TCR-Class I-restricted cytotoxicity [35], and activate cytotoxic CD8^+^ T cells by pushing naïve T cells differentiation into effector cells [36]. Heating could enhance Fas-L promoter activity and its mRNA expression in activated T cell lines, and amplify Fas-L-mediated cytotoxicity simultaneously, dependent upon thermal activation of HSF1 [37].

### Exosomes

Tumor-cell derived exosomes are recognized as potential immunostimulatory factors, due to containing large amounts of tumor antigens [38]. Exosomes from heated tumor cells carry and present tumor antigens to APCs, activate DCs and induce tumor-specific CD8^+^ T cell responses [39]. Chen et al.[40] found that exosomes released from heat-stressed tumor cells contained a lot of chemokines such as CCL2-5 and CCL20 *via* lipid raft dependent pathway, which attracted DCs, CD4^+^ and CD8^+^ T cells to infiltrate into tumor mass. Furthermore, Clayton et al.[41] reported that HSP70 originated from tumor exosomes can activate NK cells selectively, leading to augmentation of an immune response. Thus, HT could contribute to stimulate anti-tumor immune responses by heating-induced exosomes.

## METHODS OF LOCAL HYPERTHERMIA

Local hyperthermia, a common heat therapy, is specially suitable for head and neck cancers because of their superficial anatomic sites. The process of local heating is to place a contacting medium on tumor surface, then the tumor tissues were heated by antennas or applicators emitting electromagnetic wave or ultrasound. Nowadays, various antenna or applicator types have been used in clinic, e.g. microwave antennas, ultrasound transducers, laser fibres, and heat sources (ferromagnetic seeds) [42]. Therefore, the methods of local HT can be divided into three major types : ultrasound therapy, microwave therapy, and near-infrared (NIR) PTT, dependent on different physical frequencies and properties.

### Ultrasound hyperthermia

Clinically, 1-MHz ultrasound is frequently used, and the maximum penetration depth is about 7~8 cm. But it is not suitable for the presence of bone and air-tissue interfaces in the treatment region [12]. Clinical hyperthermia treatments for head and neck tumors showed that the temperature distribution could be highly modified by adjusting the power to individual rings while holding the transducer stationary [43]. Tu et al. [44] reported that the best simulated temperature distributions were produced by bidirectionally scanned, 2 MHz, f number 2.0ultrasoundtransducers whose powers were modulated as a function of position. The simulated temperature distributions from such modulated bidirectional scans were significantly better than those of both unidirectional and unmodulated bidirectional scans.

### Microwave hyperthermia

The frequency of microwave HT ranges from 430 to 2450 MHz, and the higher frequency, the more shallow tissue penetration. Commercial heating devices representative use 430 MHz, 915 MHz, and 2.45 GHz [45]. And the maximum penetration depth is about 3~4 cm if used 915 MHz microwave clinically [12]. From simulation studies of head and neck hyperthermia, Rijnen et al. [46] imposed the required positioning accuracy to be within ±5 mm, and the water bolus shape, and stability and skin contact have an important impact on treatment quality. This redesign will help to improve not only treatment quality and reproducibility, but also patient comfort and operator handling.

### Near-Infrared Photothermal Therapy (PTT)

**Figure 2:**
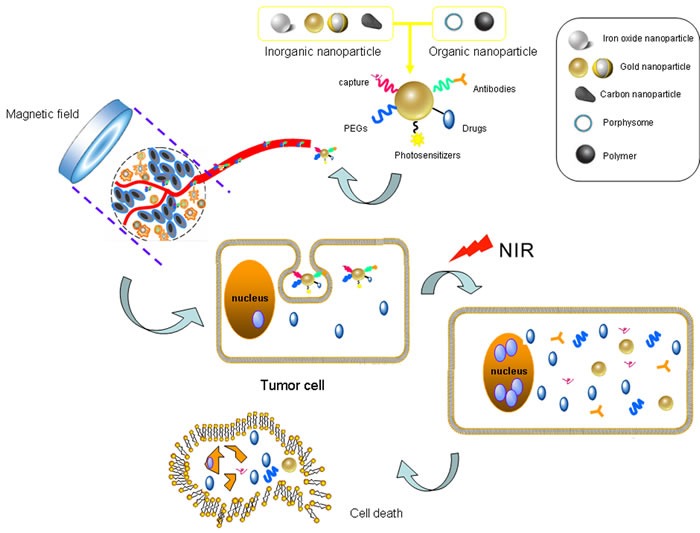
The near-infrared photothermal therapy (PTT) employs nanoparticles to kill tumor cells *via* light-induced-heating Gold nanoparticles with multiple surface modifications are injected and recruited into tumor region guided by magnetic field and active targeting function. Tumor cells swallow these nanoparticle compounds accompanied by the loaded drugs. Near-infrared (NIR) light provides energy which are converted into heat by gold nanoparticles, and boosts tumor cell death in coordination with targeted chemotherapy.

PTT employs the assistant of light-absorbing photothermal agents to treat tumors by light-induced-heating (Figure 2) [47]. This therapy requests PTT agents to convert light at certain wavelengths into heat to harm cancer cells by thermally induced necrosis [48]. Thus, PTT has the potential to better the specificity of HNSCC treatment through localizing laser irradiation and improving the accumulation of PTT agents, and minimizing comorbidities to surrounding healthy tissues.

The laser power threshold for the photothermal destruction of cells after the nanoparticle treatment is found to be 20 times lower than that required to destroy HSC oral cancer cells in the normal PTT, that is, without nanoparticles [49]. El-Sayed et al. [50] found that the malignant cells (two oral squamous carcinoma cell lines HSC 313andHOC 3 Clone 8) require less than half the laser energy to be killed than the benign cells after incubation with anti-EGFR antibody conjugated Au nanoparticles, which indicated that PTT using anti-EGFR conjugated Au nanoparticles was efficient in oral squamous carcinoma. Another report found that the EGFR-targeted Pc 4-nanoformulation was preferentially taken up by EGFR-positive H&N SCC-15 cells and showed a significant anti-tumor effect*in vitro*and*in vivo* [51]. At present, nanoparticle (NP)-enabled near-infrared photothermal therapy as a sophisticated approach of HT treatment has been widely gained attention.

## CLINICAL APPLICATION OF LOCAL HYPERTHERMIA IN HEAD AND NECK CANCER

### Local HT of primary cancer

Hyperthermia combined with radiotherapy has improved clinical response, local control, and survival in numerous phase II studies and several randomized trials for patients with head and neck cancers. A total of 56 locally advanced head and neck cancer patients, without metastatic disease, were randomized to radiation therapy arm alone or radiation-hyperthermia arm. Complete response (CR) was 42.4% of radiation group comparing to 78.6% in radiation-HT [52]. Jones et al. [53] showed that, in a prospective randomized clinical trail, one hundred twenty-two patients with superficial tumors (3 cm depth) were enrolled to partly receive a test dose of HT. The CR rate was 66.1% in HT combined with radiotherapy group and 42.3% in radiotherapy group. Kouloulias group [54] showed that 60% of the patients with 20 superficial tumors, including submandibular lymph nodes from head and neck cancers, had a complete response and microwave heating should be over 44 °C for favourable treatment response, when combined with radiotherapy.

And, hyperthermia combined with chemotherapy also has bettered clinical response for patients with head and neck cancers. Tohnai et al. [55, 56] reported that eight patients with primary cancer of the oral cavity were preoperatively treated by combined treatment with hyperthermia and chemotherapy. As a result, CR was observed in seven patients and partial response (PR) in one, and postoperative pathological examination showed no residual tumor cells in any specimen. Our group found the thermochemotherapy, a combination of local HT with 915 MHz microwave heating and chemotherapy, cured nearly all lower lip squamous cell carcinoma without metastases, and achieved excellent cosmetic and functional preservation [57].

Hyperthermia plus radiotherapy and chemotherapy, thermochemoradiation therapy, was shown as a nice therapy for 31 patients with primary laryngeal cancer (T3-4N0-3M0). Radiotherapy was given, local heat was given twice a week, and chemotherapy was performed in the beginning part of each stage of treatment. For the whole group CR was 25 (80.6%), partial response was 6 (19.3%). Five-year OR was 88.2% for T3N0, 3~62.1% for T4N1 [58]. Hoshina et al. [59] have applied thermochemotherapy combined with radiotherapy to 25patients with advancedand/or recurrentheadandneckcancers and radiochemotherapy without hyperthermia for 22 patients. There was a significant difference in the CR and total response rate, and the local control rate between these two groups, which confirmed that thermochemoradiotherapy is an effective strategy for patients with advanced head and neck cancers.

In 2014, Takeda et al. [60] showed that a combination of dendritic cell therapy andhyperthermiawas beneficial for the 14 patients with advanced or recurrenthead and neck cancer. These findings indicated that immunological therapy combined with HT might be regarded as a novel therapeutic agent for head and neck cancer clinically.

### Local HT with recurrence or metastasis cancer

Local hyperthermia was also clinically applied to treat the recurrence or metastasis of head and neck tumors. Gabriele et al. [61] enrolled 14 patients with recurrent head and neck cancers, and all patients had previously undergone radiotherapy (20~60 Gy), while HT was operated at 434 MHz/45~75W. Forty-five days later they observed 40% CR, 13.3% PR. And patients with cervical lymph nodes metastases were randomized into two groups: hyperthermia group (76/154) and control group (78/154). The addition of microwave hyperthermia to radiotherapy and cisplatin chemotherapy significantly increased the 3-month and 5-year CR rates, and 3, 5-year OR rates in the HT group. Therefore, hyperthermia combined with chemoradiotherapy for the treatment of cervical lymph node metastasis of nasopharyngeal carcinoma is an effective therapy [62]. Additionally, whether heating enhancing side-effects of the chemotherapy agents remains an open question. A phase I trial to assess the safety of arsenic trioxide in 11 patients with advanced or recurrent head and neck cancer, who were treated with radiation and hyperthermia, yet not finding the evidence of amplificatory toxicities due to radiation or hyperthermia [63].

Advanced oralcancerpatients with N3 cervical lymph node metastases are particularly difficult to treat and have a poor prognosis. Valdagni et al. [64] found that radicalirradiationplustwice a week local HT significantly enhanced the chance of early local control ofN3 necknodeswithout exhibiting an increase of acute local toxicity in 44N3metastatic squamous cell cervical lymph-nodes. Serin et al. [65] have reported their study about twenty-one patients with recurrent and metastatic head and neck carcinomas were treated with radiation, cisplatin and ultrasound hyperthermia in combination, and come to a conclusion that the trimodality treatment is effective, but still need improvement, such as offering better heating at depth. Nine patients with N3 cervical lymph node metastases ofOSCC underwent thermochemoradiation therapy using super selective intra-arterial infusion with docetaxel and cisplatin. Treatment consisted of hyperthermia, superselective intra-arterial infusions and daily concurrent radiation therapy for 4-6 weeks. The result showed that during follow-up, 5 patients were alive without disease. Five-year survival and locoregional control rates were 51% and 88%, respectively [66]. An 80-year-old female of squamous cell carcinoma of the tongue with advanced N3 cervical lymph node metastases was treated with a combination of radiotherapy, super selective intra-arterial chemotherapy and four sessions ofhyperthermiafor cervical lymph node metastases. The patient had shown no clinical or radiological evidence of local recurrence or distant metastases 6 years after the end of treatment [67].

However, some experiments showed that HT cannot better the prognosis of head and neck cancers. In 1995, the results of a phase III, clinical trial of local microwave hyperthermia and megavoltage radiation in the treatment of 145 naturally occurring canine head and neck cancers showed that there was no significant difference in best tumor response nor patient survival between the two treatment groups [68]. Further randomized studies with bettering sample size and methodology are necessary to obtain a definite conclusion in the future.

## NANOPARTICLE-BASED HYPERTHERMIA IN HEAD AND NECK CANCER

Conventional hyperthermia methods do not thermally discriminate between the target and the surrounding normal tissues, and this non-selective tissue heating can lead to serious side effects. Recently, With the development of nanomaterials, nanotechnology provides a novel and original solution for this disadvantage, and nanoparticle-based medicine application represents a novel breakthrough in revolutionizing to tumor diagnosis and treatment in head and neck cancer. The materials of nanoparticles can generally be divided into two types: inorganic nanoparticle and organic nanoparticle. Inorganic nanoparticles, including metal nanomaterials and carbon nanomaterials, have been proved to have great potential in targeted hyperthermic therapy [69, 70]. Organic nanoparticles, such as porphysome and light-absorbing conductive polymers, are increasingly concerned. The following focuses on several typical nanoparticles.

### Iron oxide nanoparticles

Iron oxide nanoparticles (mainly Fe_3_O_4_ and its conjugates), approximately 100 nm or smaller in size, can serve as hyperthermic agent due to Brownian or Neél modes, and carry active substance for cancer treatment. The advantages of minimal toxicities, potential for rapid heating, and perdurable stability greatly increase their popularity [71]. And they can also be detected by conventional imaging techniques (such as MRI and PET), and have been used for ‘theranostic’ purposes [72, 73].

The Fe_3_O_4_@mSiO_2_ nanocarrier, consisting of a Fe_3_O_4_ nanoparticle core and a mesoporous silica (mSiO_2_) shell, were able to load the anti-cancer drug doxorubicin and control drug release by the magnetic hyperthermia of Fe_3_O_4_ under alternating magnetic field *in vitro*. This shows enormous potential for cancer treatment in targeted-control drug release combined hyperthermia together [74]. Magnetic iron oxide nanoparticles have been applied in local hyperthermia for treatment of head and neck cancer (Tu212 cell line) in mouse xenograft models. The tumor center temperature had dramatically elevated from room temperature to about 40°C rapidly and pathological studies showed epithelial tumor cells destruction associated with HT [75].

As a new Fe_3_O_4_ nanoparticles, the superparamagnetic iron oxide nanoparticles (SPIONs) can be used individually or associated with chemotherapy and immunotherapy. Lindemann et al. [76] evaluated the biocompatibility of superparamagnetic iron oxide nanoparticles (SPIONs), their impact on biological properties, and their cellular uptake in HNSCCs. The decreased cell proliferation in response to increased SPION concentrations was observed, suggesting that UL-D SPIONs are a promising tracer material for use in HNSCCs. In twenty rabbits bearing VX2 tumor in pyriform sinuses, randomly divided into hyperthermia group under the alternating magnetic field and control group after USPIO (Ultra-small superparamagneticiron oxide) MR scanning, Wang et al. [77] showed that USPIO indirect lymphography could localize the metastatic lymph nodes for hyperthermia and make the metastatic cervical lymph nodes apoptosis through regulating Bcl-2/Bax protein expression. Another report confirmed that the delivery of a PTT agent Pc 4 by iron oxide nanoparticles could enhance treatment efficacy and reduce PDT drug dose. The targeted IO-Pc 4 NPs have great potential to serve as both a magnetic resonance imaging (MRI) agent and PDT drug in the clinic of HNSCC [78].

However, iron nanoparticles require extremely high concentrations to achieve designated thermal enhancement, which may lead to the destruction of normal cells surrounding the tumor sometimes [79]. Besides, when patients with head and neck cancer were treated by iron nanoparticles, metallic implants, teeth's amalgam fillings and metal crowns have to be replaced by ceramics [3].

### Gold nanoparticles

Due to intensively enhanced absorption in the NIR regions, gold nanoparticles (GNPs) have been regarded as one of the most successful hyperthermic agents in nano-materials. They are small enough to penetrate widely, preferentially accumulate on tumor sites, and bind many proteins and drugs [80]. And they have been shown to improve sensitivity to radiotherapy [81, 82]. The size of GNPs, ranging from 1~100nm, may affect physicochemical parameters including cellular uptake, diffusion, cytotoxicity and efficiency of photothemal conversion. The larger the particles are, the smaller the photothermal conversion efficiencies become [83]. There are various shapes of gold nanoparticles, such as gold nanospheres, gold nanorods, gold nanoshells, and gold nanocages [84].

Recent advances have demonstrated that gold nanoshells, consisting of silica cores and gold coating, can absorb and convert NIR light to heat with an obviously efficacy and stability, due to their adjustable peak absorption wavelength tailoring the diameter-to-shell ratio, and the thinner the shell thickness, the longer the peak absorption wavelengths they become. The adjustability of gold nanoshells enables their use as strong absorbers or scatterers of NIR light in which optical penetration through tissue is optimal [85, 86]. Gold-nanoshell-loaded macrophages, following near-infrared exposure of head and neck carcinoma cells and brain tumors, significantly reduced the cell viability and suppressed tumor growth, respectively [87, 88]. Wang et al. [89] substantiated a new type gold nanoshell (AuNSs) drug delivery system (DOX-TSMLs-AuNSs-PEG), based on doxorubicin (DOX)-loaded thermosensitive magnetoliposomes (TSMLs) was successfully combined with multifunctions, such as RF-triggered controlled release, hyperthermia intensifier and MRI contrast agent. The results of DOX-TSMLs-AuNSs-PEG *in vitro* and *in vivo* indicated that the complex of gold nanoparticle is a promising effective drug delivery and target system for diagnosis and treatment of tumors. Compared to the single photodynamic therapy (PDT) or PTT, the rose Bengal-gold nanorods with combined PDT-PTT capabilities provide better therapeutic effects against oralcancerand have large potential incancertreatment [90]. Further, macrophages have been shown to be regarded as a delivery vector of gold nanoshell forphotothermalenhancement of the effects of PTT on squamous cell carcinoma *in vitro*[91, 92].

There are still many questions for the routine clinical use of gold nanoparticles in PTT, such as indeterminable acute or long-term toxicities, inprecise quantifying and prediction of the light exposure, uneven heat distribution in the treatment volume and undefined specific targeting of tumor cells [93].

### Organic nanoparticles

Organic nanoparticles, an important PTT agents, have received much attention in recent years. Polypyrrole (PPy), an organic conductive polymer, was first reported for photothermal treatment of cancer by Liu's group, owing to the outstanding stability and strong NIR absorbance. They synthesized poly (vinyl alcohol) (PVA)-coated PPy nanoparticles, injected them into mouse tumor models and obtained excellent treatment efficacy with an ultralow power NIR laser irradiation [70]. Zha et al. [94] reported PPy nanoparticle complex with good colloidal stability and dispersibility, and showed a higher photothermal conversion efficiency and NIR photostability than Au nanorods. Both *in vitro* and *in vivo* studies indicated that the organic nanoparticles could be an excellent candidate agent for PTT cancer therapy. Thermosensitive liposomes (TSLs) nanoparticles also showed wonderful image guidance and anticancer drug delivery in near-infrared laser-induced thermal therapy, resulted in relatively good efficacy in the treatment of murine xenograft tumors [95].

Lovell et al. [96] reported that porphysomes, nanovesicles formed from self-assembled porphyrin bilayers, demonstrated the multimodal potential of organic nanoparticles for biophotonic imaging and therapy. Porphysome nanoparticles, directly incorporated with Mn^3+^ ions, were capable of maintaining high conversion efficiency of NIR light into heat in PTT, while simultaneously imparting MRI sensitivity, improving photostability, and reducing toxicity of inorganic nanocrystals [97]. In the rabbit and hamster models of head and neck tumors, compared with surgery, porphysome-enabled photothermal therapy more completely eradicate primary tumors and metastatic regional lymph node while sparing the adjacent critical structures' function [98]. Further, Muhanna et al. [99] found that the multimodal porphyrin lipoprotein- mimickingnanoparticle (PLP), as a multimodal imaging and therapy platform, could enhance HNC diagnosis by integrating PET/computed tomography and fluorescence imaging, and improve head and neck cancer therapeutic efficacy and specificity by tailoring treatment *via* fluorescence-guided surgery and PDT.

Photothermal nanoparticles can convert near-infrared light into heat [100]. NIR light and nanoparticles have nontoxicity, favorable biocompatibility, and superexcellent chemical inertness, which ensure the safety and effect of photothermal therapy [101]. Beik et al. [102] extensively examined and compared four modern nanotechnology-based hyperthermia methods, such as Nano-photo-thermal therapy (NPTT) and nano-magnetic hyperthermia (NMH), nano-radio-frequency ablation (NaRFA) I, and nano- ultrasound hyperthermia (NUH).

## CONCLUSIONS

There are now strong experiment data and clinical results for applying local HT in head and neck cancer. The majority of studies all showed significant increases in complete response and/or overall survival after local HT, although occasional one or two publications have not bettered the curative effect. Although the technology of local HT in most of these studies provided only limited thermal dose control, and the devices only allowed treatment of target regions close to the skin for some decades, with the emerging and development of PTT, nanoparticle materials, and HYPERcollar3D, local HT will overcome the inaccuracy of temperature, position and dose control during the clinical application. The most obvious advantage of nanoparticles is their strongly enhanced near-infrared light absorption, especially noble metal nanoparticles, and higher orders of magnitude of acceleration compared to conventional laser phototherapy agents [103]. Paulides et al. [104] focused microwave heating combined with 3D patient-specific electromagnetic and thermal simulations for conformal, reproducible and adaptive hyperthermia application. The clinical implementation and validation of 3D guided deep hyperthermia with the HYPERcollar, and its second generation, i.e. the HYPERcollar3D, have been done. Hence, all of these technologies and approaches may have promising prospect to provide the non-invasive dose control and maximize treatment outcome of local HT in head and neck cancer in the future.
